# Pharmaceutical Lexicon

**Published:** 1873-11

**Authors:** 


*Pharmaceutical Lexicon. A Dictionary of Pharmaceutical Science. Containing a concise Explanation of the various Subjects and Terms of Pharmacy.* By H. V. Sweringen. Philadelphia: Lindsay & Blackiston, 1873. Buffalo: T. Butler & Son. By subscription only. Cloth $6.00, sheep $7.00.

The work under consideration is doubtless the result of much labor and study, and may supply a want in the literature of the Medical and Pharmaceutical profession. Many of the definitions and tables are admirable, and much information can be obtained from them. Some of the definitions do not do justice either to the subject, or the writer, and the consideration of many topics which would naturally be included in a volume of this character is entirely omitted. The publication of a second edition will afford an opportunity to revise the work: and render it a valuable book of reference for the Physician and Pharmaceutist. The printing and binding is done in the well known thorough manner of the publishers, leaving nothing to be desired.

